# HP1 induces ferroptosis of renal tubular epithelial cells through NRF2 pathway in diabetic nephropathy

**DOI:** 10.1515/biol-2022-0678

**Published:** 2023-08-08

**Authors:** Chuanqiang Zhou, Min Wu, Gaolun Liu, Li Zhou

**Affiliations:** Department of Nephrology, The First People’s Hospital of Longquanyi District, Chengdu & West China Longquan Hospital, Sichuan University, No. 669, Donglang Road, Longquanyi District, Chengdu, Sichuan Province 610100, China; Department of Nephrology, West China Hospital, Sichuan University, Chengdu, Sichuan Province 610100, China

**Keywords:** diabetic nephropathy, ferroptosis, HP1, NRF2

## Abstract

The aim of this study was to investigate the role of ferroptosis in diabetic nephropathy (DN) and the mechanism of its regulatory genes. HK-2 cells were cultured with high glucose and mice were intraperitoneally injected with streptozotocin to establish DN models. GSE111154 was analyzed to identify the abnormal expression of genes associated with DN. Cell injury was evaluated through CCK-8 assay and 4′,6-diamidino-2-phenylindole/phenylindole double staining. The levels of iron, glutathione, malondialdehyde, urinary albumin, and urinary creatinine were determined by ELISA. Furthermore, western blot and RT-qPCR were used to detect protein and mRNA levels, respectively. Our data showed that heterochromatin protein 1 is an abnormally elevated gene related to DN and is further elevated by ferroptosis activators. Inhibition of HP1 significantly inhibited ferroptosis but promoted cell viability. In addition, nuclear factor erythroid2-related factor2 (NRF2) was decreased in DN cell model, but increased under the action of ferroptosis activators. NRF2 silencing reversed the protective effects of HP1 inhibition on HK-2 cells. Additionally, HP1 silencing also alleviated kidney damage in DN mice. Collectively, these findings suggest that inhibiting HP1 inhibits ferroptosis via NRF2 pathway, thereby protecting renal tubular epithelial cells from damage.

## Graphical abstract

**Figure:**
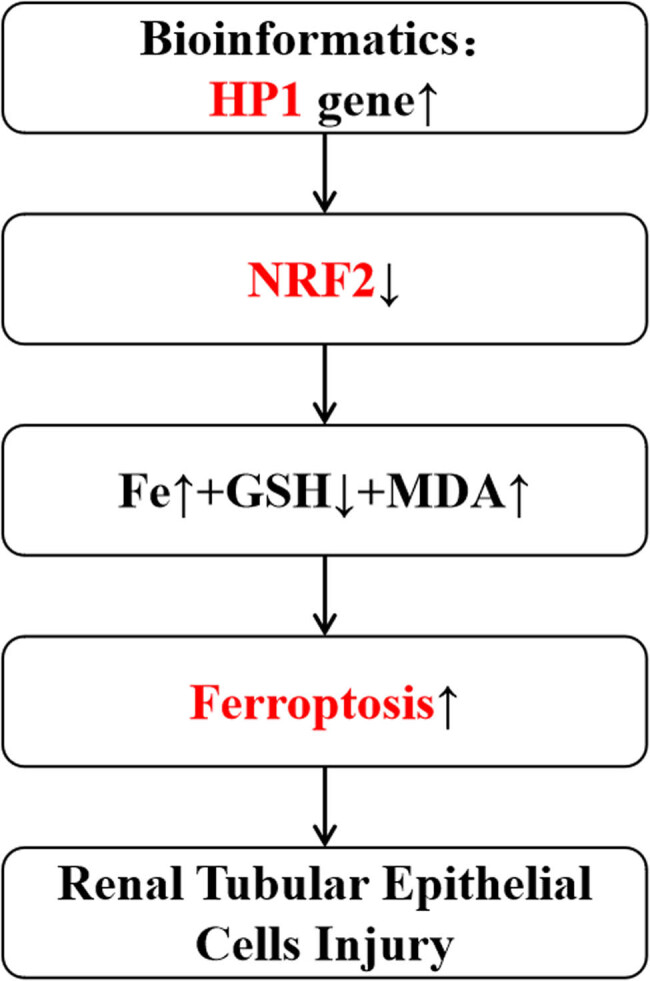

## 1 Introduction

Diabetic nephropathy (DN) is a serious microvascular complication of diabetes and the most common cause of end stage renal disease. At present, the clinical treatment of DN cannot effectively interfere with the occurrence process of DN [1]. Therefore, it is of great theoretical significance and practical application value to find the key molecules involved in the DN process and apply them in the prevention and treatment of DN. Early DN is usually characterized by albuminuria caused by glomerular dysfunction, glomerular sclerosis, and glomerular filtration barrier breakdown. Renal tubular epithelial cell injury plays an important role in the occurrence and development of DN [2,3].

Ferroptosis is a kind of lipid peroxidation and excessive oxidative stress of unsaturated fatty acids caused by iron ion deposition, which leads to the destruction of the selective permeability of plasma membrane and ultimately leads to cell death. Iron plays a very important role in human life activities, and a lot of evidence shows that iron metabolism is closely related to DN. Iron metabolism indexes such as iron, ferritin, and transferrin are closely related to the occurrence and development of DN [4,5]. Therefore, it is crucial to understand the pathogenesis of DN and take appropriate intervention measures to inhibit ferroptosis during the course of DN.

The nuclear transcription factor erythroid 2-related factor 2 (NRF2) is located on human chromosome 2q31.2 and encodes a leucine zipper structure transcription factor, which is an important endogenous antioxidant factor in the cell. NRF2 can promote the expression of Glutathione peroxidase 4 (GPX4) and inhibit the occurrence of ferroptosis. Recently, many DN-related genes have been found to regulate ferroptosis in DN by regulating the NRF2 pathway [6,7]. Therefore, it is important to search for genes regulating NRF2 pathway in DN.

In this study, DN-related genes were identified, and whether DN-related genes could modulate ferroptosis through NRF2 pathway was studied *in vitro* and *in vivo*. This study aims to provide an additional treatment option for DN.

## 2 Materials and methods

### 2.1 Microarray data

The human gene expression dataset GSE111154 consisting of four non-diabetic samples and four DN samples were downloaded from NCBI Gene Expression Omnibus. Genes with *p* < 0.05 and |logFC| > 2 were filtrated.

### 2.2 Cell culture and grouping

Human renal tubular epithelial cells HK-2 from American Type Culture Collection were cultured in RPMI-1640 medium (Gibco) containing 10% fetal bovine serum (Gemini) and 1% antibiotics (100 U/mL penicillin and 100 μg/mL streptomycin). After the cells grew and fused to 60%, they were inoculated on six-well plates and randomly divided into normal glucose group (5.5 mM glucose [8], control), high glucose stimulated group (30 mM glucose [8], HG), si-NC transfection group (HG + si-NC), si-heterochromatin protein 1 (HP1) transfection group (HG + si-HP1), si-NRF2 transfection group (HG + si-HP1 + si-NRF2), and its negative control group (HG + si-HP1 + si-NC). In the control group and HG group, the medium was discarded with a cell confluence of 70%, and normal medium and high-glucose medium were added, respectively. Cells were transfected with corresponding plasmids. The transfection steps were as follows: solution 1 for transfection (125 μg serum-free antibiotic-free medium + 5 μL lipo3000 + 2.5 μg siRNA) and solution 2 for transfection (125 μg serum-free antibiotic-free medium + 3.75 μL p3000) were mixed and then added in culture medium. After 12 h of transfection, the transfection efficiency was detected by RT-qPCR, and then high-glucose medium was added, respectively. The supernatant was collected after culturing for 48 h, and the relevant indicators were detected. Cell viability of each group was evaluated by CCK-8 assay. BAY-61-3606 (2 μM, Selleck) as an apoptosis inhibitor was used to treat HK-2 cells [9]. Erastin (an inducer of ferroptosis, Selleck, 10 μM), RSL3 (an inducer of ferroptosis, Selleck, 0.1 μM), Ferrostatin-1 (Fer-1; a ferroptosis inhibitor, Selleck, 1 μM), and Liproxstatin-1 (Lip-1; a ferroptosis inhibitor, Selleck, 200 nM) were used to modulate ferroptosis of HK-2 cells [8,10,11,12].

### 2.3 Construction and processing of animal models

C57BL/6 N 8-week-old mice were purchased from Beijing Baiaosaitu Company. All mice were raised in the Animal Experimental Center of The First People’s Hospital of Longquanyi District. The mice were fed under controlled environmental conditions (22℃, light and dark cycle for 12 h), and divided into four groups of six: negative control group (NC), DN group, HP1 knockdown negative control group (DN + sh-NC), and HP1 knockdown group (DN + sh-HP1). The control group was fed with normal diet, and the other three groups received high-fat diet and intraperitoneal injection of streptozotocin 50 mg/kg (consecutively for 5 days) to induce DN 4 weeks later [7]. NC group was injected with the same amount of normal saline. shRNA was designed according to HP1 sequence, and shRNA fragments were synthesized from Shanghai SunnyBio company. 2 μg sh-HP1 plasmid and 4 μg packaged plasmid were co-transfected into 293T cells to package adenovirus. 48 h later, the supernatant medium containing the virus was collected and purified by centrifugation to harvest adenovirus. The mice of DN + sh-NC group and DN + sh-HP1 group were, respectively, injected with 0.1 mL of PBS containing 3 × 10^8^ PFU empty adenovirus vector (sh-NC) or sh-HP1 adenovirus through the tail vein, and the same dose of sh-NC or sh-HP1 adenovirus was repeated through the vein 2 weeks later. Four weeks after the injection of the adenovirus, the mice were killed by the cervical dislocation method after the abdominal injection of 0.3% pentobarbital sodium (10 mL/kg). Blood samples from their tail veins were collected for fasting glucose level measurement using a glucose analyzer (Sinocare). After the model was established, mice in each group were fed for 12 weeks and their blood glucose was measured. Mice with a blood glucose level of ≥16.7 mmol/L were considered to be successful models of DN. The body weight of mice was weighed and blood and urine samples were collected. Urine samples were collected in a metabolic cage, in which mice were free to eat and drink, and urine volume was monitored.

### 2.4 Collection of renal tissue

Mice were anesthetized by intraperitoneal injection of 10% chloral hydrate (0.35 mL/100 g, Shanghai Macklin Biochemical Co., Ltd) to collect the renal tissues. After laparotomy, 0.9% normal saline was injected into the left ventricle, and the perfusion was stopped when the liver and kidney of mice turned white. The kidney tissues were partially preserved with 4% paraformaldehyde.


**Ethical approval:** The research related to animal use has been complied with all the relevant national regulations and institutional policies for the care and use of animals.

### 2.5 Hematoxylin-eosin (HE) staining

The renal tissue fixed in paraformaldehyde was dehydrated with alcohol and embedded in paraffin wax to make 4 μm thick tissue sections, which were stained by HE, and the pathological changes of kidney were observed under light microscope (CX23, Olympus).

### 2.6 CCK-8 assay

HK-2 cells in different groups were seeded in a 96-well plate with 5 × 10^3^ cell/well. The CCK-8 solution (10 μL, Beyotime) was added to the culture plate and incubated for another 1 h at 37℃. A microplate Reader (Multiscan EX) was executed to examine the optical density at 450 nm wavelength.

### 2.7 4′,6-diamidino-2-phenylindole/phenylindole (DAPI/PI) double staining

DAPI/PI double staining was performed to evaluate cell death [13]. HK-2 cells in each group were centrifuged to discard the supernatant, and cells were suspended with 1 mL cell staining buffer, followed by 5 μL DAPI staining solution (Beyotime) and 5 μL PI staining solution (Beyotime). The cells were placed in a dark environment at 4℃ and stained for 30 min. The cells were thoroughly cleaned with PBS two times before cell smears were prepared and observed with fluorescence microscope (TE2000, Nikon, Japan).

### 2.8 ELISA

The tissues and cells were lysed and added to the iron determination buffer (Iron Colorimetric Assay Kit, Leagene), then centrifuged to obtain the supernatant for determination according to the manufacturer’s instructions. The experimental buffer was added to the 96-well plate, incubated at room temperature for 0.5 h, and mixed reagents were added and incubated in the dark for 15 min. The absorbance at 562 nm was measured by a microplate reader (Multiskan Spectrum 51119570; Thermo Fisher). For glutathione (GSH) and malondialdehyde (MDA) contents, the levels were measured according to the manufacturer’s instructions using commercial kits (Sigma-Aldrich). Urine samples were centrifuged for 20 min and the supernatant was collected. The concentrations of urinary albumin and urinary creatinine were detected by ELISA kits (Beyotime). The urinary albumin/creatinine ratio (ACR) were then calculated.

### 2.9 RT-qPCR

Cells and tissues were mixed with TRIzol® reagent (Thermo Fisher Scientific) to extract total RNA. Reverse transcription and qPCR were carried out using a BlazeTaq One-Step SYBR Green RT-qPCR Kit (QP071, GeneCopoeia) on a SEDI Thermo Cycler controlled by Control Bus Net software package (Wealtec Bioscience). All primers were designed and synthesized by Nanjing GenScript Biotech Co., Ltd, and GAPDH was used as internal reference. Fold changes in the indicated genes were calculated using the 2^−ΔΔCt^ method [14]. The primer sequences were as follows:

HP1: (Forward): 5′-CTAGACAGGCGCGTGGTTAAG-3′;

(Reverse): 5′-GCTCAGGGCAATCCAAGTTTT-3′;

IGHA1: (Forward): 5′-GCAGCATTCGGATTCACATTC-3′;

(Reverse): 5′-GATGTTCCTGATGTTGTCTCTGG-3′;

IGLC7: (Forward): 5′-GTTTCCGCTAGTGGGACATCA-3′;

(Reverse): 5′-CTGACCTCTGTGTCGAATGTG-3′;

NFR2: (Forward): 5′-TCAGCGACGGAAAGAGTATGA-3′;

(Reverse): 5′-CCACTGGTTTCTGACTGGATGT-3′;

FTH1: (Forward): 5′-CAAGTGCGCCAGAACTACCA-3′;

(Reverse): 5′-ACAGATAGACGTAGGAGGCATAC-3′;

GPX4: (Forward): 5′-TGTGCATCCCGCGATGATT-3′;

(Reverse): 5′-CCCTGTACTTATCCAGGCAGA-3′;

TFR1: (Forward): 5′-GTTTCTGCCAGCCCCTTATTAT-3′;

(Reverse): 5′-GCAAGGAAAGGATATGCAGCA-3′;

GAPDH: (Forward): 5′-AGGTGAAGGTCGGAGTCAACG-3′;

(Reverse): 5′-AGGGGTCATTGATGGCAACA-3′;

### 2.10 Western blot assay

RIPA reagent (Sigma-Aldrich) was used to extract proteins from cells and tissues. Protein concentration was determined using a BCA kit (Sigma-Aldrich). Additionally, proteins (20 µg/lane) were separated by 15% SDS-PAGE and then transferred to PVDF membranes (Bio-Rad). The membranes were blocked with 5% skim milk for 2 h. Membranes were then incubated with primary antibodies, including GPX4 (ab125066, 1:2,000, Abcam), NRF2 (ab62352, 1:500, Abcam), p-NRF2 (ab76026, 1:2,000, Abcam), and anti-GAPDH (ab8245, 1:500, Abcam) at 4°C overnight, followed by incubation with secondary antibody (ab6721, 1:2,000, Abcam) for 1 h. Subsequently, the membrane was stained with an ECL western blotting kit (ab193759, Abcam). Finally, protein bands were visualized using an ECL system (Thermo Fisher Scientific).

### 2.11 Luciferase reporter assay

The plasmids containing the wild-type NRF2 (NRF2-wt) and mutant NRF2 (NRF2-mut) were purchased from RiboBio. Plasmids were co-transfected into HK-2 cells. The luciferase activity was assessed with a Double-Luciferase Reporter Assay Kit (Promega) via the Dual-Light Chemiluminescent Reporter Gene Assay System (Berthold), which was normalized to firefly luciferase activity.

### 2.12 Statistical analysis

SPSS v.19.0 (IBM) was used for statistical analyses. All experiments were performed in triplicate and all data are presented as mean values ± SD. Differences between two groups were evaluated using Student’s *t*-test and one-way ANOVA for multiple groups. Statistical significance was set at *p* < 0.05.

## 3 Results

### 3.1 HP1 is a ferroptosis-related gene in DN

**Figure 1:**
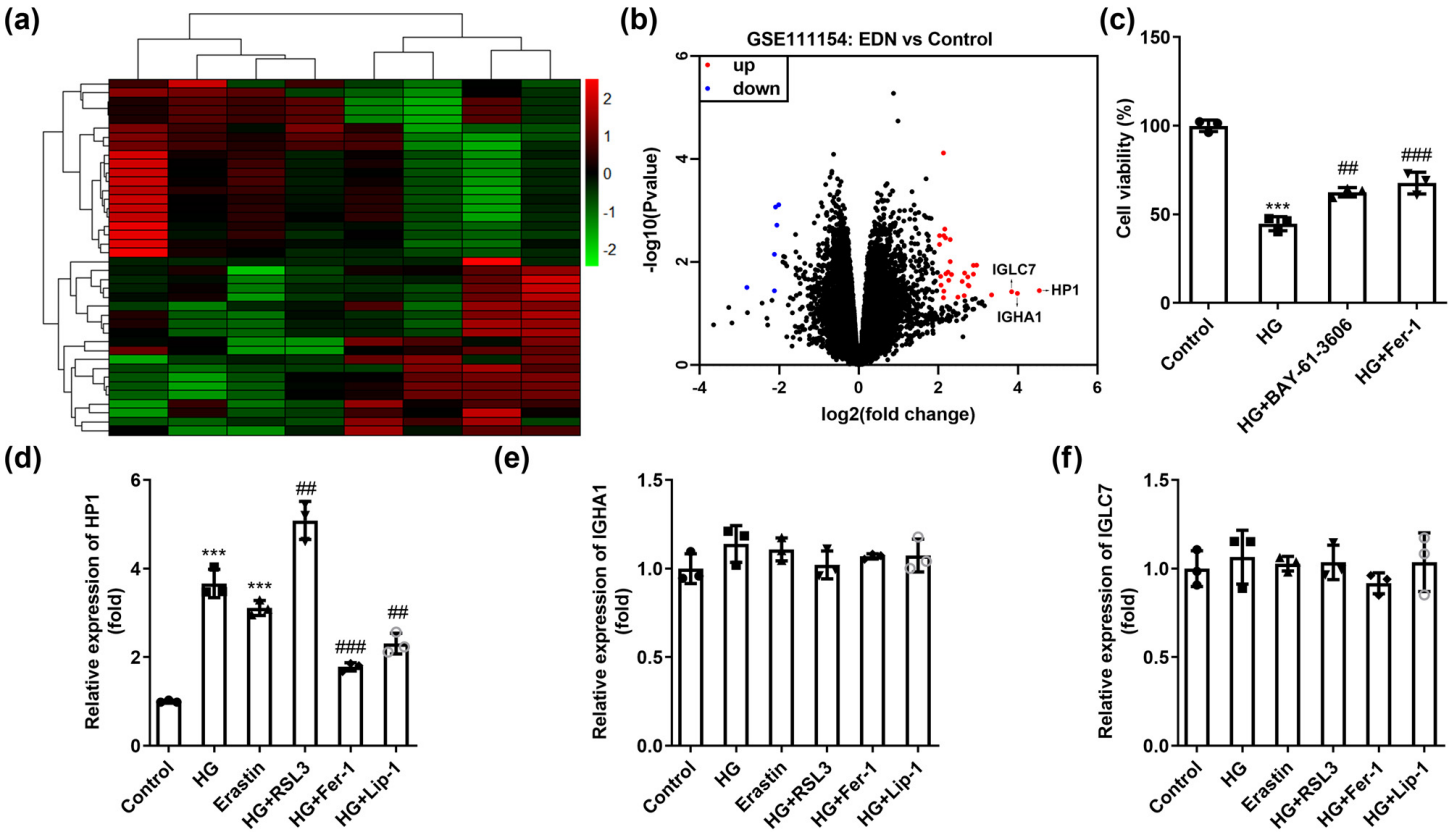
HP1 is a ferroptosis-related gene in DN. (a) The heat map and (b) volcano map of DN-related gene data obtained from GSE111154. (c) Cell viability of HK-2 cells evaluated by CCK-8 assay under the treatment of high glucose, an apoptosis inhibitor, and a ferroptosis inhibitor. (d–f) The first three genes (HP1, IGHA1, and IGLC7) with the most significant upregulation were evaluated in DN model cells under the induction of different ferroptosis agonists or inhibitors. ***p < 0.001 (vs control), ^##^ *p* < 0.01, ^###^ *p* < 0.001 (vs HG).

First, the microarray GSE111154 was analyzed, and the heat map illustrated that there are a large number of differentially expressed genes among the four pairs of DN samples (Figure 1a). Under the criterion of *p* < 0.05 and |logFC| > 2, 6 downregulated genes and 31 elevated ones were identified (Figure 1b). Then, three top upregulated genes (HP1, IGHA1, and IGLC7) were selected as candidate genes. As shown in Figure 1c, HG treatment significantly inhibited the viability of HK-2 cells, while an apoptosis inhibitor BAY-61-3606 and a ferroptosis inhibitor Fer-1 significantly promoted the viability of HK-2 cells on the basis of HG treatment (Figure 1b). Therefore, we hypothesized that inhibiting ferroptosis helps protect HK-2 cells from high glucose induced injury. Subsequently, the expressions of HP1, IGHA1, and IGLC7 in DN model cells were evaluated with different ferroptosis inducers or inhibitors. Our data demonstrated that HP1 levels increased sharply after HG treatment (Figure 1d), while IGHA1 (Figure 1e) and IGLC7 (Figure 1f) levels were not statistically different among groups. In addition, Erastin, a ferroptosis activator, also increased HP1, and RSL3 further increased HP1 on the basis of HG. Conversely, ferroptosis inhibitors Fer-1 and Lip-1 both inhibited HP1 levels on the basis of HG. In summary, HP1 may be related to ferroptosis in DN *in vitro*.

### 3.2 Inhibition of HP1 suppresses ferroptosis induced by HG treatment

**Figure 2:**
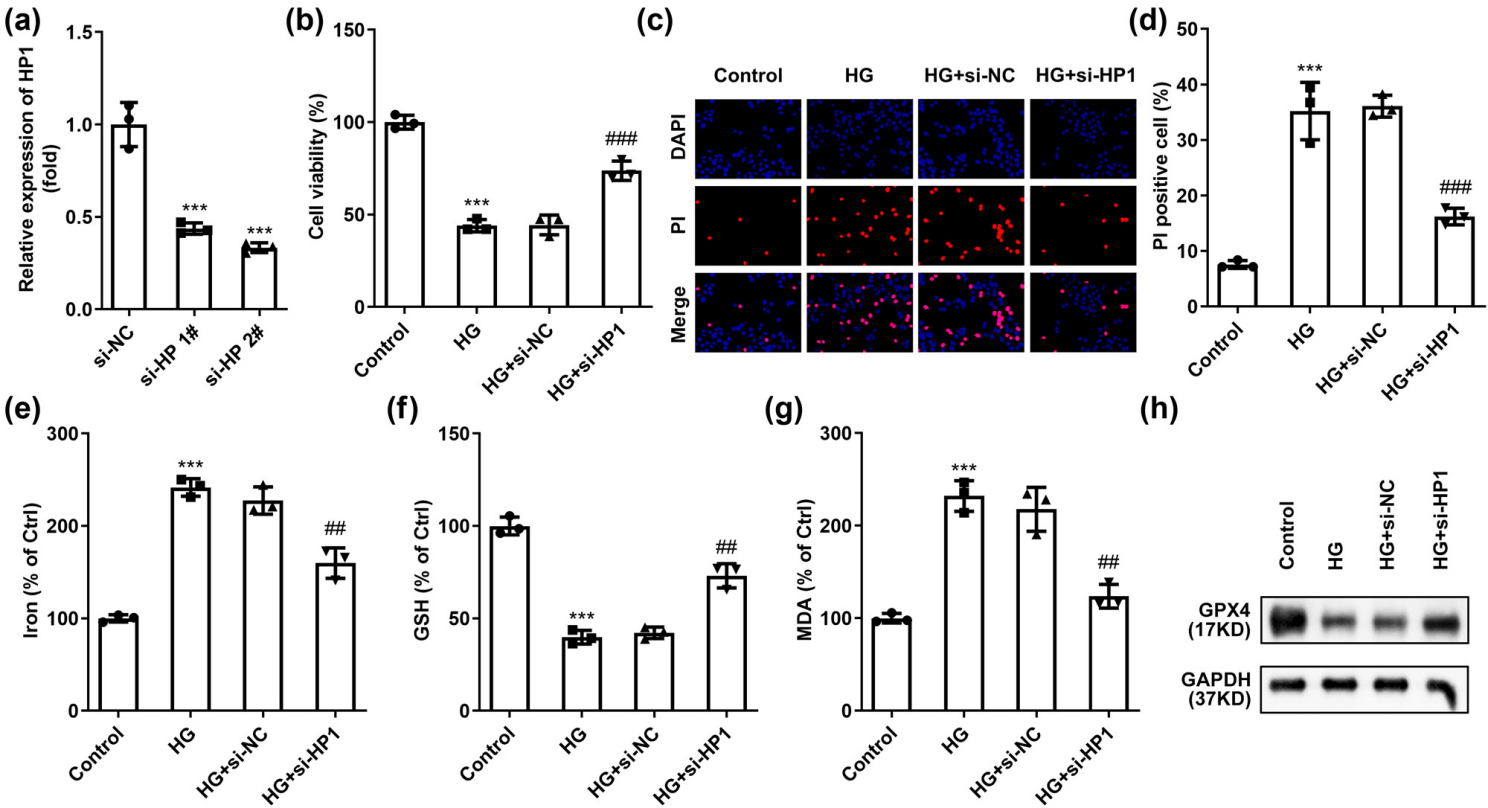
Inhibition of HP1 suppresses ferroptosis induced by HG treatment. (a) HP1 level was validated using RT-qPCR after transfection. (b) Cell viability of HG treated HK-2 cells determined by CCK-8. (c) Cell apoptosis of HK-2 cells under different treatments was determined by DAPI/PI staining. (d–g) Iron, MDA, and GSH levels were combined to evaluate ferroptosis. (h) Representative western blot images of GPX4 protein. ***p < 0.001 (vs si-NC or control), ^##^ *p* < 0.01, ^###^ *p* < 0.001 (vs HG + si-NC). Western blot analysis was performed three times.

To determine the role of HP1 in HG-induced ferroptosis in HK-2 cells, we reduced the levels of HP1 and verified this using RT-qPCR (Figure 2a). HP1 was inhibited in HK-2 cells, and the inhibition was more potent in si-HP 2# group. si-HP 2# plasmid was selected for subsequent experiments (hereinafter referred to collectively as si-HP). HG treatment significantly reduced cell viability (Figure 2b) and induced apoptosis (Figure 2c). Meanwhile, ferroptosis of HK-2 cells was also promoted, which was manifested as a significant increase in iron and MDA levels, and a significant decrease in GSH levels (Figure 2d–g). However, by reversing the levels of the above indicators, HP1 silencing significantly reversed the effects of HG on HK-2 cells.

### 3.3 HP1 regulates HG-induced ferroptosis via NRF2 signaling pathway

**Figure 3:**
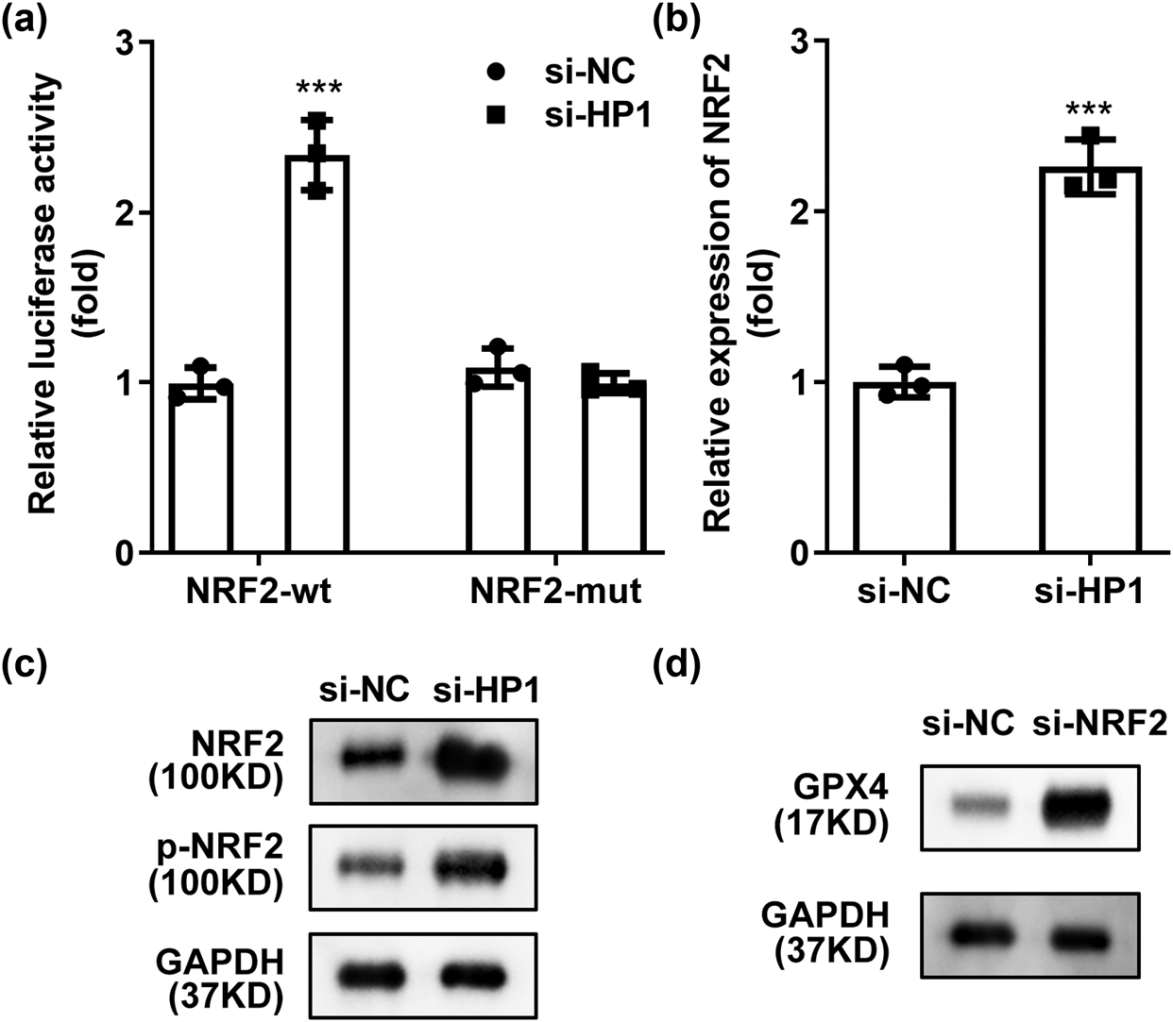
NRF2 binds to HP1. (a) Luciferase reporter assay was conducted to evaluate binding relationship between NRF2 and HP1. (b) NRF2 level was determined by RT-qPCR after IGHA1 knockdown. (c) Protein level of NRF2 and (d) GPX4 were evaluated by western blot assay. ***p < 0.001. Western blot analysis was performed three times.

**Figure 4:**
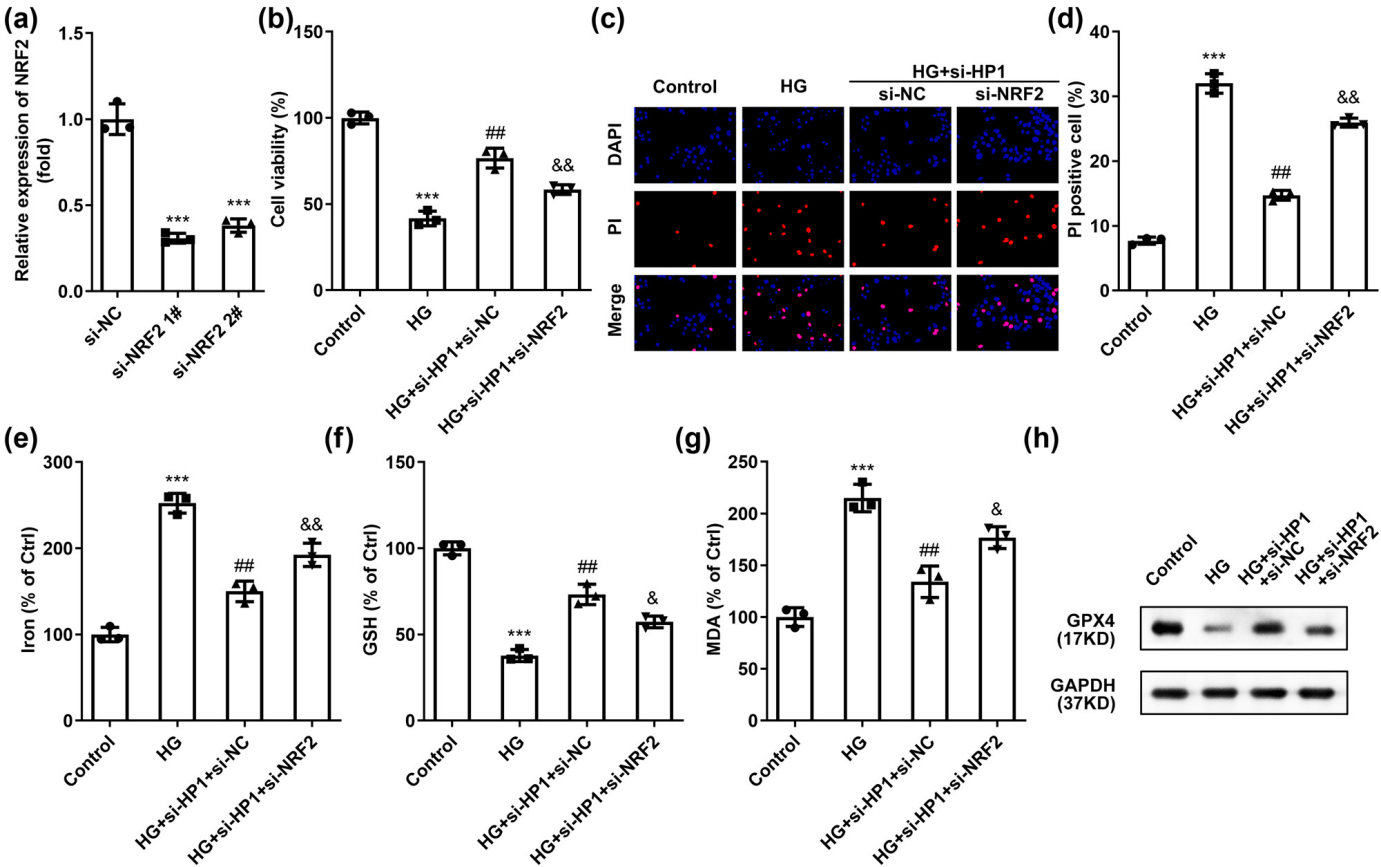
HP1 regulates HG-induced ferroptosis via NRF2 signaling pathway. (a) NRF2 level was validated using RT-qPCR after transfection. (b) Cell viability of HG treated cells determined by CCK-8. (c) Cell apoptosis of HK-2 cells under different treatment was determined by DAPI/PI staining. (d–g) Iron, MDA, and GSH levels were combined to evaluate ferroptosis. (h) Representative western blot images of GPX4 protein. ***p < 0.001 (vs si-NC or control), ^##^ *p* < 0.01 (vs HG), ^&^ *p* < 0.05, ^&&^ *p* < 0.01 (vs HG + si-HP1 + si-NC). Western blot analysis was performed three times.

According to previous studies, HP1 and histone methyltransferase G9a can form a certain transcriptional inhibitory complex, regulating the transcriptional activity of target genes [15], and NRF2 is a key factor in ferroptosis [16]. Therefore, we speculated that HP1 inhibits the transcriptional activity of NRF2 and reduces its expression level. As shown in Figure 3a, the luciferase activity in NRF2-wt group was significantly upregulated by HP1 knockdown. However, these effects were not observed in the mutated NRF2 groups, suggesting that NRF2 binds to HP1 (Figure 3a). At the same time, while inhibiting HP1, NRF2 mRNA levels increased in HK-2 cells (Figure 3b). Protein level of NRF2 and phosphated NRF2 were also increased by knockdown of HP1 (Figure 3c). In addition, inhibition of NRF2 in HK-2 cells significantly induced the GPX4 protein expression (Figure 3d). NRF2 was then inhibited in HK-2 cells, more effectively in si-NFR2 1# group (Figure 4a). si-NFR2 1# plasmid was selected for subsequent experiments (hereinafter referred to collectively as si-NFR2). As expected, knockdown of NRF2 dramatically reversed the effects of si-HP1 on cell viability (Figure 4b), cell apoptosis (Figure 4c), and ferroptosis (Figure 4d–g).

### 3.4 Knockdown of HP1 alleviated renal injury of DN mice by suppressing ferroptosis

**Figure 5:**
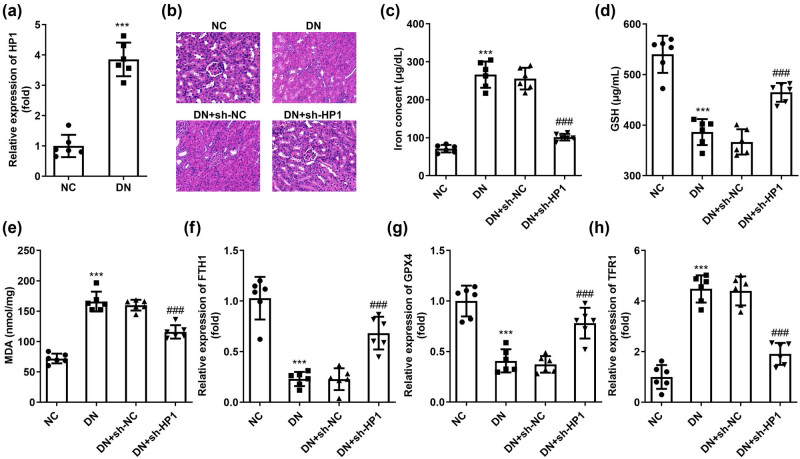
Knockdown of HP1 suppresses ferroptosis *in vivo*. (a) HP1 levels in renal tissue of DN mice were evaluated using RT-qPCR. (b) HE staining assay of renal tissue of DN mice was performed to observe the pathological morphology. (c–e) Iron, MDA, and GSH levels were combined to evaluate ferroptosis. (f–h) mRNA expression of FTH1, GPX4, and TFR1 in HK-2 cells with different treatments. ***p < 0.001 (vs NC), ^###^ *p* < 0.001 (vs DN + sh-NC).

**Figure 6:**
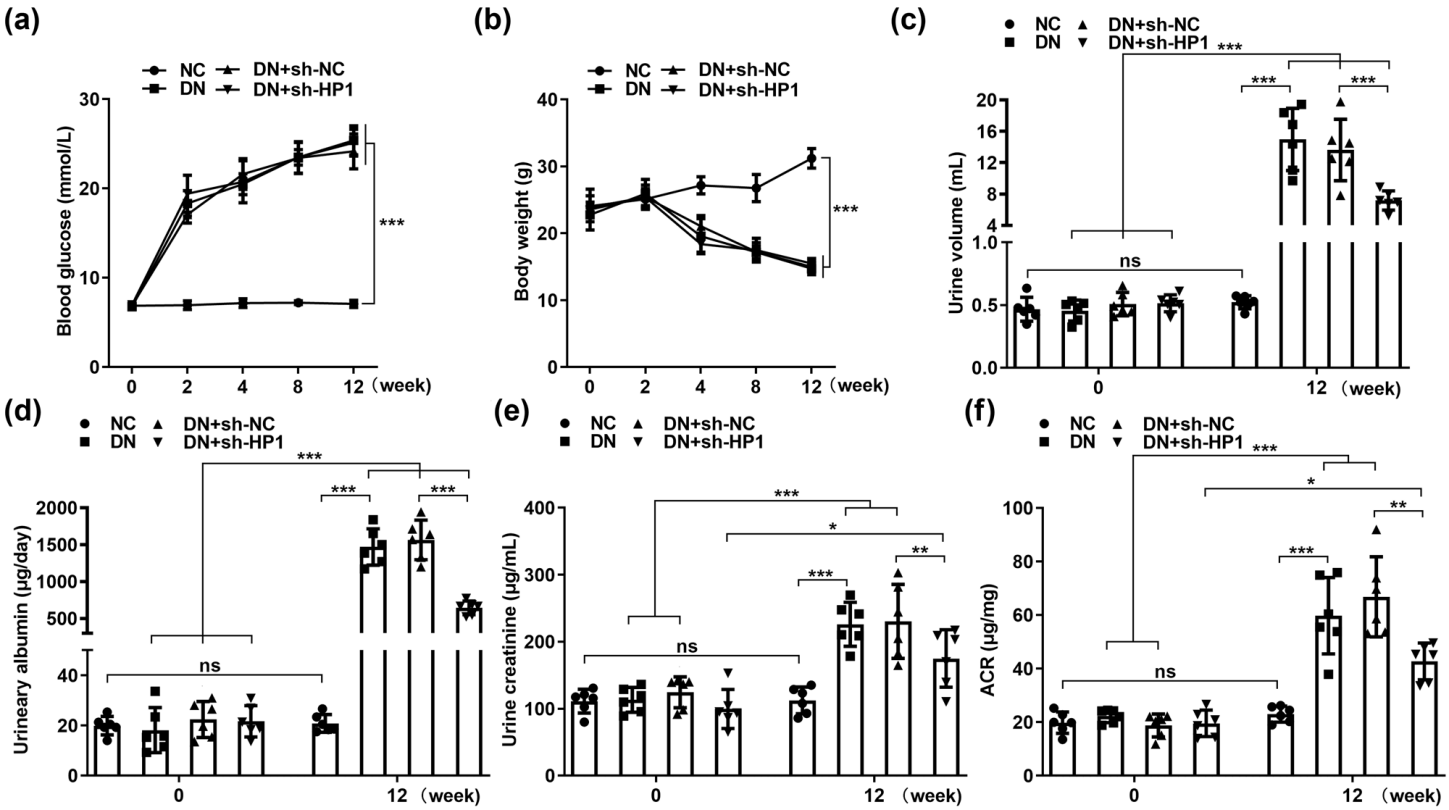
Knockdown of HP1 alleviated renal injury of DN mice by suppressing ferroptosis. (a) Blood glucose, (b) body weight, (c) urine volume, (d) urinary albumin, (e) urinary creatinine, and (f) ACR ratio were evaluated in DN groups with different treatments. *p < 0.05, **p < 0.01, ***p < 0.001.

Subsequently, the role of HP1 in *in vivo* experiments was investigated. Compared with the negative control group, the level of HP1 in DN model group was also significantly upregulated (Figure 5a). In addition, we observed the pathological morphology of renal tissue of mice in each group, and the results of HE staining showed that the renal tissue of mice in the NC group had clear structure, regular shape, and no obvious lesions. Glomerular atrophy, renal tubule dilation, and interstitial fibrosis were observed in the DN model group. The renal tissue morphology of sh-NC group had no significant change compared with DN group. After knocking down HP1, the damage degree of kidney tissue was significantly improved compared with that of DN model group (Figure 5b). DN-induced iron (Figure 5c) and MDA (Figure 5e), and DN-reduced GSH (Figure 5d) were all prominently reversed by HP1 silencing. Furthermore, both protein and mRNA levels of ferritin heavy chain 1 and GPX4 reduced in DN mice were dramatically increased, and elevated transferrin receptor 1 (TFR1) in DN mice was decreased under the situation of HP1 silencing (Figure 5f–h). After DN mice were established, we found that blood glucose levels were prominently higher in the three DN groups than the control group (Figure 6a). In contrast, body weight was prominently lower in the three DN groups than the control group (Figure 6b). Moreover, the urine volume (Figure 6c), urinary albumin (Figure 6d), urinary creatinine (Figure 6e), and ACR ratio (Figure 6f) were all dramatically elevated 12 weeks later in the three DN groups, while in the control group, there was no statistical difference in each indicator after 12 weeks.

## 4 Discussion

Glomerular endothelial injury is an important feature of inflammatory response and for the occurrence and development of DN, and ferroptosis is a cell death that is closely related to inflammatory response [17,18]. Current work is focused on the role of ferroptosis and the modulation mechanism. Our findings demonstrated that HP1 silencing protected glomerular injury of DN by modulating ferroptosis *in vitro* and *in vivo* through NRF2 pathway.

Ferroptosis is dependent on iron and oxidative stress for the regulation of cell death, which differs morphologically, biochemistry, and genetically from apoptosis, necrosis, and autophagy [16]. Ferroptosis is a non-apoptotic, non-necrotic regulator of cell death without rapid ATP depletion, and inhibitors of apoptosis, autophagy, or necrosis cannot suppress ferroptosis [16]. Overall, ferroptosis leads to cell death through iron-mediated accumulation of lipid ROS, which interferes with cell integrity and membrane fluidity and permeability [8,9,10]. Furthermore, since iron-dependent oxidative stress and lipid peroxidation are common features of ferroptosis and inflammatory diseases, ferroptosis has been intensively studied in a variety of inflammatory diseases [19]. For instance, CD8(+)T cells released interferon gamma to inhibit glutamate-cystine antiporter system xc-, enhance lipid peroxidation and to promote ferroptosis in tumor cells [20]. Ferroptosis also plays a regulatory role in Alzheimer’s disease, Parkinson’s disease, cerebral hemorrhage, and other diseases [19]. In addition, butyrate disrupted iron homeostasis by activation of NCOA4-mediated ferritinophagy, leading to ferroptosis in periodontitis [21]. Hence, we speculated that inhibiting ferroptosis in the course of DN may be a potential therapeutic direction.

At present, reports on pathways related to ferroptosis in DN involve VEGF/Akt/ERK, NLRP3, and NRF2 pathways [22,23,24,25]. Among these pathways, NRF2 is a central regulator that regulates cellular oxidative stress response and maintains intracellular redox homeostasis. NRF2 can activate the transcription of various antioxidant genes (such as NQO1, HO-1), maintain the oxidative homeostasis in the body, and then reduce the damage to cells and tissues caused by oxidative stress [26]. A large number of research works have reported that NRF2 can significantly improve mitochondrial dysfunction and inhibit ferroptosis [27]. Notably, many proteins and enzymes that prevent lipid peroxidation are target genes of NRF2, such as the ferroptosis key protein GPX4 [28]. Furthermore, studies have shown that inhibition of NRF2 activity enhances the sensitivity of colorectal cancer to the ferroptosis inducer RSL3 [29]. In non-small cell lung cancer, NRF2 can regulate the expression of oxidative stress target genes and iron metabolism proteins, thereby affecting the anticancer activity of ferroptosis inducer Erastin, and oxidative stress may be involved in the coordinated regulation of ferroptosis and apoptosis [30,31]. Therefore, NRF2 is not only a key transcription factor responsible for maintaining cellular metabolism, redox, and protein balance but also plays a pivotal role in the regulation of the ferroptosis pathway. Therefore, significantly activating NRF2 expression genes may significantly alleviate renal damage caused by DN.

Currently, our data suggested that HP1 binds to NRF2, and NRF2 could prominently reverse the effects of HP1 on modulating HG-induced ferroptosis. HP1, an evolutionarily conserved protein encoded by CBX5 gene, has multiple functions in heterochromatin formation, gene regulation, and mitotic progression, and forms a complex network of gene, RNA, and protein interactions [32]. HP1 is a DN-related gene, and its regulatory role was investigated for the first time in this study. Inhibition of HP1 prominently suppressed ferroptosis induced by HG treated HK-2 cells. Furthermore, inhibition of HP1 was also found to alleviate renal injury induced by HG treatment *in vivo*.

There are some limitations in this study, i.e., whether HP1 regulates other ferroptosis-related pathways and potential molecular players in DN needs to be further investigated.

## 5 Conclusion

Taken together, HP1 knockdown protected against high-glucose induced renal tubular injury by inhibiting ferroptosis related to NRF2 pathway. HP1 was first found to be associated with the regulation of ferroptosis in DN. Therefore, HP1 inhibition may represent an alternative therapeutic target for the treatment of DN.

## References

[1] Yamazaki T, Mimura I, Tanaka T, Nangaku M. Treatment of diabetic kidney disease: current and future. Diabetes Metab J. 2021;45(1):11–26. 10.4093/dmj.2020.0217 PMC7850867 33508907

[2] Hou Y, Wang S, Jiang L, Sun X, Li J, Wang N, et al. Patulin induces acute kidney injury in mice through autophagy-ferroptosis pathway. J Agric Food Chem. 2022;70(20):6213–23. 10.1021/acs.jafc.1c08349 35543324

[3] Yang T, Hu Y, Chen S, Li L, Cao X, Yuan J, et al. YY1 inactivated transcription co-regulator PGC-1α to promote mitochondrial dysfunction of early diabetic nephropathy-associated tubulointerstitial fibrosis. Cell Biol Toxicol. 2023;39(2):391–413. 10.1007/s10565-022-09711-7 35445903

[4] Wang Y, Bi R, Quan F, Cao Q, Lin Y, Yue C, et al. Ferroptosis involves in renal tubular cell death in diabetic nephropathy. Eur J Pharmacol. 2020;888:173574. 10.1016/j.ejphar.2020.173574 32976829

[5] Kim S, Kang SW, Joo J, Han SH, Shin H, Nam BY, et al. Characterization of ferroptosis in kidney tubular cell death under diabetic conditions. Cell Death Dis. 2021;12(2):160. 10.1038/s41419-021-03452-x PMC7870666 33558472

[6] Wu Y, Zhao Y, Yang H, Wang Y, Chen Y. HMGB1 regulates ferroptosis through Nrf2 pathway in mesangial cells in response to high glucose. Biosci Rep. 2021;41(2):1. 10.1042/BSR20202924 PMC7897919 33565572

[7] Zhang Q, Hu Y, Hu J, Ding Y, Shen Y, Xu H, et al. Sp1-mediated upregulation of Prdx6 expression prevents podocyte injury in diabetic nephropathy via mitigation of oxidative stress and ferroptosis. Life Sci. 2021;278:119529. 10.1016/j.lfs.2021.119529 33894270

[8] Li S, Zheng L, Zhang J, Liu X, Wu Z. Inhibition of ferroptosis by up-regulating Nrf2 delayed the progression of diabetic nephropathy. Free Radic Biol Med. 2021;162:435–49. 10.1016/j.freeradbiomed.2020.10.323 33152439

[9] He X, Huang Y, Liu Y, Zhang X, Yue P, Ma X, et al. BAY613606 attenuates neuroinflammation and neurofunctional damage by inhibiting microglial Mincle/Syk signaling response after traumatic brain injury. Int J Mol Med. 2022;49(1):5. 10.3892/ijmm.2021.5060 PMC8612304 34751408

[10] Hu Z, Zhang H, Yi B, Yang S, Liu J, Hu J, et al. VDR activation attenuates cisplatin induced AKI by inhibiting ferroptosis. Cell Death Dis. 2020;11(1):73. 10.1038/s41419-020-2256-z PMC6989512 31996668

[11] Li D, Liu B, Fan Y, Liu M, Han B, Meng Y, et al. Nuciferine protects against folic acid-induced acute kidney injury by inhibiting ferroptosis. Br J Pharmacol. 2021;178(5):1182–99. 10.1111/bph.15364 33450067

[12] Gao Z, Zhang Z, Gu D, Li Y, Zhang K, Dong X, et al. Hemin mitigates contrast‐induced nephropathy by inhibiting ferroptosis via HO‐1/Nrf2/GPX4 pathway. Clin Exp Pharmacology Physiol. 2022;49(8):858–70. 10.1111/1440-1681.13673 35598290

[13] Das V, Kaishap PP, Duarah G, Chikkaputtaiah C, Deka BH, Pal M. Cytotoxic and apoptosis-inducing effects of novel 8-amido isocoumarin derivatives against breast cancer cells. Naunyn Schmiedebergs Arch Pharmacol. 2021;394(7):1437–49. 10.1007/s00210-021-02063-9 33649978

[14] Pan J, Zhao L. Long non-coding RNA histone deacetylase 4 antisense RNA 1 (HDAC4-AS1) inhibits HDAC4 expression in human ARPE-19 cells with hypoxic stress. Bioengineered. 2021;12(1):2228–37. 10.1080/21655979.2021.1933821 PMC8806694 34057022

[15] Pokorna P, Krepl M, Sponer J. Residues flanking the ARK(me3)T/S motif allow binding of diverse targets to the HP1 chromodomain: Insights from molecular dynamics simulations. Biochim Biophys Acta Gen Subj. 2021;1865(1):129771. 10.1016/j.bbagen.2020.129771 33153976

[16] Wei R, Zhao Y, Wang J, Yang X, Li S, Wang Y, et al. Tagitinin C induces ferroptosis through PERK-Nrf2-HO-1 signaling pathway in colorectal cancer cells. Int J Biol Sci. 2021;17(11):2703–17. 10.7150/ijbs.59404 PMC8326123 34345202

[17] Belavgeni A, Meyer C, Stumpf J, Hugo C, Linkermann A. Ferroptosis and necroptosis in the kidney. Cell Chem Biol. 2020;27(4):448–62. 10.1016/j.chembiol.2020.03.016 32302582

[18] Sun Y, Chen P, Zhai B, Zhang M, Xiang Y, Fang J, et al. The emerging role of ferroptosis in inflammation. Biomed Pharmacother. 2020;127:110108. 10.1016/j.biopha.2020.110108 32234642

[19] Mao H, Zhao Y, Li H, Lei L. Ferroptosis as an emerging target in inflammatory diseases. Prog Biophys Mol Biol. 2020;155:20–8. 10.1016/j.pbiomolbio.2020.04.001 32311424

[20] Liao P, Wang W, Wang W, Kryczek I, Li X, Bian Y, et al. CD8(+) T cells and fatty acids orchestrate tumor ferroptosis and immunity via ACSL4 cancer Cell. 2022;40(4):365–78. 10.1016/j.ccell.2022.02.003 PMC9007863 35216678

[21] Zhao Y, Li J, Guo W, Li H, Lei L. Periodontitis-level butyrate-induced ferroptosis in periodontal ligament fibroblasts by activation of ferritinophagy. Cell Death Discov. 2020;6(1):119. 10.1038/s41420-020-00356-1 PMC7655826 33298848

[22] Tan H, Chen J, Li Y, Li Y, Zhong Y, Li G, et al. Glabridin, a bioactive component of licorice, ameliorates diabetic nephropathy by regulating ferroptosis and the VEGF/Akt/ERK pathways. Mol Med. 2022;28(1):58. 10.1186/s10020-022-00481-w PMC9123664 35596156

[23] Wang X, Li Q, Sui B, Xu M, Pu Z, Qiu T. Schisandrin A from Schisandra chinensis attenuates ferroptosis and NLRP3 inflammasome-mediated pyroptosis in diabetic nephropathy through mitochondrial damage by AdipoR1 ubiquitination. Oxid Med Cell Longev. 2022;2022:5411462. 10.1155/2022/5411462 PMC9391610 35996380

[24] Wang W, Jiang X, Gao C, Chen Z. Salusin‑β participates in high glucose‑induced HK‑2 cell ferroptosis in a Nrf‑2‑dependent manner. Mol Med Rep. 2021;24(3):674. 10.3892/mmr.2021.12313 PMC8335735 34296310

[25] Feng Q, Yang Y, Qiao Y, Zheng Y, Yu X, Liu F, et al. Quercetin ameliorates diabetic kidney injury by inhibiting ferroptosis via activating Nrf2/HO-1 signaling pathway. Am J Chin Med. 2023;51(4):997–1018. 10.1142/S0192415X23500465 37046368

[26] Lim JO, Song KH, Lee IS, Lee SJ, Kim WI, Pak SW, et al. Cimicifugae rhizoma extract attenuates oxidative stress and airway inflammation via the upregulation of Nrf2/HO-1/NQO1 and downregulation of NF-kappaB phosphorylation in ovalbumin-induced asthma. Antioxid (Basel). 2021;10(10):1626. 10.3390/antiox10101626 PMC8533435 34679759

[27] Wang X, Chen X, Zhou W, Men H, Bao T, Sun Y, et al. Ferroptosis is essential for diabetic cardiomyopathy and is prevented by sulforaphane via AMPK/NRF2 pathways. Acta Pharm Sin B. 2022;12(2):708–22. 10.1016/j.apsb.2021.10.005 PMC8897044 35256941

[28] Ge MH, Tian H, Mao L, Li DY, Lin JQ, Hu HS, et al. Zinc attenuates ferroptosis and promotes functional recovery in contusion spinal cord injury by activating Nrf2/GPX4 defense pathway. CNS Neurosci Ther. 2021;27(9):1023–40. 10.1111/cns.13657 PMC8339532 33951302

[29] Yang J, Mo J, Dai J, Ye C, Cen W, Zheng X, et al. Cetuximab promotes RSL3-induced ferroptosis by suppressing the Nrf2/HO-1 signalling pathway in KRAS mutant colorectal cancer. Cell Death Dis. 2021;12(11):1079. 10.1038/s41419-021-04367-3 PMC8590697 34775496

[30] Gai C, Yu M, Li Z, Wang Y, Ding D, Zheng J, et al. Acetaminophen sensitizing erastin-induced ferroptosis via modulation of Nrf2/heme oxygenase-1 signaling pathway in non-small-cell lung cancer. J Cell Physiol. 2020;235(4):3329–39. 10.1002/jcp.29221 PMC13482668 31541463

[31] Gai C, Liu C, Wu X, Yu M, Zheng J, Zhang W, et al. MT1DP loaded by folate-modified liposomes sensitizes erastin-induced ferroptosis via regulating miR-365a-3p/NRF2 axis in non-small cell lung cancer cells. Cell Death Dis. 2020;11(9):751. 10.1038/s41419-020-02939-3 PMC7490417 32929075

[32] Keenen MM, Brown D, Brennan LD, Renger R, Khoo H, Carlson CR, et al. HP1 proteins compact DNA into mechanically and positionally stable phase separated domains. Elife. 2021;10:e64563. 10.7554/eLife.64563 PMC7932698 33661100

